# Characterization of variations within the rumen metaproteome of Holstein dairy cattle relative to morning feed offering

**DOI:** 10.1038/s41598-020-59974-5

**Published:** 2020-02-21

**Authors:** Mallory C. Honan, Sabrina L. Greenwood

**Affiliations:** Department of Animal and Veterinary Sciences, The University of Vermont, 570 Main Street, Burlington, VT 05405 USA

**Keywords:** Proteomics, Proteomic analysis

## Abstract

Few studies have utilized proteomic techniques to progress our knowledge of protein-mediated pathways within the rumen microbial community, and no previous research has used these techniques to investigate the patterns or variations of these proteins within this community. It was hypothesized that there would be fluctuations of rumen microbial protein abundances due to feed intake-mediated nutrient availability and that these could be identified using non gel-based proteomic techniques. This study investigated the fluctuations of bovine rumen metaproteome utilizing three mid to late-lactation Holsteins. Rumen fluid was collected at three timepoints on three days relative to their first morning feed offering (0 h, 4 h, and 6 h). Samples were pooled within timepoint within cow across day, analyzed using LC-MS/MS techniques, and analyzed for variations across hour of sampling using PROC MIXED of SAS with orthogonal contrasts to determine linear and quadratic effects. A total of 658 proteins were characterized across 19 microbial species, with 68 proteins identified from a variety of 15 species affected by time of collection. Translation-related proteins such as 50S and 30S ribosomal protein subunit variants and elongation factors were positively correlated with hour of sampling. Results suggest that as nutrients become more readily available, microbes shift from conversion-focused biosynthetic routes to more encompassing DNA-driven pathways.

## Introduction

The pregastric rumen is the dominant site of microbial colonization and microbe-mediated fermentation within the ruminant digestive tract, and functionality of this chamber is a key factor that dictates the animal’s efficiency of nutrient utilization and production^1–4^. Feed and production efficiency, ruminant animal health, and environmental emissions are all affected by rumen ecology, hence there is growing interest within the ruminant livestock sector to understand the *in situ* or *vivo* functionality within and among rumen microbes. Current knowledge of the rumen microbiome is cross-disciplinary and rapidly expanding, with novel research emerging that is focused on diversity analysis and community structures of the microbiota^5–8^, as well as metabolic pathway analysis and metatranscriptomics^9–12^.

Despite advances in our understanding, there is still a gap in knowledge regarding the undercurrents and interplay of microbe-specific metabolic pathways because of their dynamicity, adaptability, and complexity. Utilizing a variety of approaches to characterize the rumen in terms of microbial ecology and pathway dynamics appears to be necessary. For instance, microbial diversity analysis has revealed that basal diet and diurnal rumen pH patterns can be somewhat independent of bacterial community profile^13^, while more recently, Söllinger *et al*.^14^ paired metabolomics with quantitative metatranscriptomics to assess the diurnal fluctuations of individual rumen microorganisms and also reported a dissociation between the functional microbial transcripts and microbiome pathway products such as methane. Layered within these challenges, the central dogma of translation appears to be disjointed, with microbial RNA not reflective of protein abundances within the rumen, possibly due to post-translational modifications along with other adaptations^14–16^. In addition, issues such as a limitation in analytic capabilities to discern *in vivo* complexities and variations due to external drivers such as endogenous and management influences, have slowed progress and the application of knowledge to commercial systems.

Proteomic techniques are now integrated in livestock research, with published applications in milk^17–19^, urine^20–22^, plasma^23,24^, and reproductive fluid^25,26^. Proteomic characterization of the rumen metaproteome includes unique challenges due to the multitude of residing organisms, but undoubtedly would yield valuable data bearing in mind the reliance of animal production on protein-mediated pathways and microbial protein production. Only two known previous works have been published that apply proteomic techniques to investigate the protein profile of the rumen, both utilizing gel techniques which may limit the number of proteins that can be identified^15,27^. Using these techniques, Snelling and Wallace^27^ were able to identify 50 unique proteins in rumen fluid samples collected from beef cattle and lambs; however, protein identification in rumen fluid samples collected from grazing dairy cattle was not achieved due to obstruction of protein bands on the gels by plant-based humic compounds. More recently, Hart *et al*.^15^ used gel-based techniques to examine the rumen metaproteome, and included successful techniques to partially separate interfering contaminants, including humic acid, from the protein extract. Both publications provided valuable solutions to methodological challenges and a first glimpse of microbe-specific proteins in the rumen. Combining these protein isolation techniques with newer isobaric labeling methods was hypothesized to be a feasible approach to broaden the scope of rumen metaproteome characterization. It was further hypothesized that there would be fluctuations in rumen microbial protein abundances due to feed intake-mediated nutrient availability in lactating dairy cattle. The objectives of this experiment were to use non gel-based fractionation methods and isobaric labeling techniques to further the characterization of the rumen metaproteome within Holstein dairy cattle and identify whether variations in these profiles relative to first morning feed offering could be elucidated using this approach.

## Results

Metaproteomic analysis using the outlined protocol resulted in identification of 698 proteins across 19 microbial species including multiple strains (see Supplementary Table S1 for list of proteins identified within species and Supplementary Table S2 for data files). Using Proteome Discoverer 2.2, the proteins from the following microbial species and their strains were identified: *Butyrivibrio hungatei* MB2003, *Butyrivibrio hungatei* XBD2006, *Butyrivibrio hungatei* DSM 14810, [*Eubacterium*] *cellulosolvens* 6, *Eubacterium ruminantium*, *Fibrobacter succinogenes* (strain ATCC 19169/S85), *Lactobacillus ruminis* ATCC 27782/RF3, *Megasphaera elsdenii* DSM 20460, *Methanosarcina barkeri* 3, *Oxalobacter formigenes* HOxBLS, *Ruminococcus albus* SY3, *Prevotella aff. ruminicola* Tc2-24, *Prevotella bryantii* FB3001, *Prevotella bryantii* KHPX14, *Prevotella bryantii* TC1-1, *Prevotella bryantii* B14, *Prevotella ruminicola* (*Bacteroides ruminicola*) AR32, *Prevotella ruminicola* (*Bacteroides ruminicola*) ATCC 19189, *Prevotella ruminicola* (*Bacteroides ruminicola*) BPI-162, *Prevotella ruminicola* (*Bacteroides ruminicola*) BPI-34, *Prevotella ruminicola* (*Bacteroides ruminicola*) D31d, *Prevotella ruminicola* (*Bacteroides ruminicola*) KHT3, *Prevotella ruminicola* ATCC 19189/JCM 8958/23 and ATCC 19189/JCM 8958/23, *Pseudobacteroides cellulosolvens* ATCC 35603/DSM 2933, *Pseudobutyrivibrio ruminis* ACV-9, *Pseudobutyrivibrio ruminis* JK10, *Pseudobutyrivibrio ruminis* JK626, *Pseudobutyrivibrio ruminis* DSM 9787, *Ruminococcus bromii* 5AMG, *Ruminococcus bromii* AF15–36, *Ruminococcus bromii* AF21–10LB, *Ruminococcus bromii* AF25-7LB, *Ruminococcus bromii* AM32-13AC, *Ruminococcus bromii* AM46-2BH, *Ruminococcus bromii* ATCC 27255, *Ruminococcus bromii* CF01-14, *Ruminococcus bromii* L2-36, *Ruminococcus bromii* TM09-18AC, *Ruminococcus bromii* TM09-5AC, *Ruminococcus bromii* TM10-21, *Ruminococcus bromii* YE282, *Ruminococcus bromii* L2-63, *Ruminococcus flavefaciens* 007c, *Treponema bryantii*, *Treponema saccharophilum* DSM 2985, and *Wolinella succinogenes* ATCC 29543/DSM 1740/LMG 7466/NCTC 11488/FDC 602 W. Of the 698 proteins identified across all searched species, 658 proteins were labeled and present in all samples, and these were quantified for downstream statistical analysis and bioinformatics (Supplementary Table S2). Results were grouped and interpreted based on abundance pattern shifts relative to morning feeding with LOW0 representing proteins that demonstrated an increase in abundance either quadratically or linearly relative to initial sampling (0 h) and HIGH0 representing proteins that began with a higher abundance at 0 h and had either a linear or quadratic decrease in the latter sampling points at 4 h and 6 h.

Of the quantified proteins, there were 68 proteins across 15 microbial species that were affected by time of sampling, including 88.2% that responded linearly, and 30.9% being affected by a quadratic pattern of change.

### Microbial proteins with an increase in abundance from 0 h to 4 h

There were 10 microbial species with proteins represented in this group (LOW0): *E. cellulosolvens, B. hungatei, P. aff. Ruminicola, P. bryantii, P. ruminicola, P. ruminis, R. albus, R. flavefaciens*, and *T. saccharophilum*. Of the proteins that were affected by time of sampling, 45.6% had lower abundances at 0 h compared to 4 h. As represented in Table 1, 16 of the LOW0 proteins were different variants of the 50 s ribosomal protein (L14, *R. flavefaciens*; L33, L7/12, *E. cellulosolvens*; L6, *R. albus;* L3, L14, L16, L21, L31, *P. aff. ruminicola;* L1, L16, *P. ruminicola;* L21, L22, *P. ruminis;* L4, L5, *R. bromii*; L11, *T. saccharophilum*), while shifts in abundance of individual 30 s ribosomal proteins (S11, and S5) from *E. cellulosolvens, B. hungatei, P. aff. ruminicola*, and *P. ruminicola*, totaled 6 of the proteins in LOW0 based on their abundance patterns. Elongation factor proteins of *E. cellulosolvens* (elongation factor Tu) and *P. ruminis,* and *R. bromii* (elongation factor G) were lower in abundance at 0 h compared to 4 h. Other proteins that were lower in abundance at 0 h compared to 4 h that were unique to a specific species were those from *P. ruminicola*, including starch phosphorylase, alpha-amylase, and carboxypeptidase regulatory-like domain-containing protein.

### Microbial proteins with a decrease in abundance from 0 h to 4 h

There were 12 microbial species represented in the HIGH0 group: *E*. *cellulosolvens*, *B. hungatei*, *E. ruminantium*, *F. succinogenes, M. elsdenii, M. barkeri, P. ruminicola, P. ruminis, R. albus*, *R. flavefaciens*, *T. saccharophilum,™* and *W. succinogenes*. The functional groupings of proteins that had higher abundances at 0 h compared to 4 h (54.4% of proteins affected by time of sampling) are listed in Table 1. There was a single 50 s ribosomal protein (from *E. ruminantium*) that exhibited a decrease in abundance from 0 h to 4 h.

**Table 1:** Scaled abundance values of the 68 proteins within strained rumen grab samples collected from Holstein dairy cows that were affected by time of sampling relative to morning feeding (0 h, 4 h, or 6 h after feeding). Proteins were grouped by whether they increased (LOW0) or decreased (HIGH0) in abundance either linearly or quadratically relative to 0 h sampling.

Protein	Description	Species	0 h	4 h	6 h	SE	P value linear	P value quadratic	Group
A0A1D9P0Y8	Sugar ABC transporter substrate-binding protein	Butyrivibrio hungatei	105.23	97.40	97.30	0.50	<0.0001	0.007	HIGH0
C9RNK1	DNA-binding protein HU	Fibrobacter succinogenes	121.10	89.30	89.53	3.70	0.001	0.059	HIGH0
I5ATH2	50S ribosomal protein L7/L12	[Eubacterium] cellulosolvens	91.40	104.93	103.67	1.47	0.001	0.027	LOW0
A0A1D9P1Q9	FeS cluster assembly scaffold protein NifU	Butyrivibrio hungatei	102.90	98.13	98.93	0.50	0.001	0.015	HIGH0
A0A1I0LZ55	50S ribosomal protein L21	Prevotella aff. ruminicola	93.60	102.33	104.10	1.23	0.001	0.302	LOW0
A0A1T4M509	50S ribosomal protein L27	Eubacterium ruminantium	106.37	100.03	93.57	1.39	0.001	0.252	HIGH0
A0A2G3DV35	Phosphoenolpyruvate carboxykinase (ATP)	Pseudobutyrivibrio ruminis	109.40	96.03	94.53	1.77	0.001	0.169	HIGH0
D5EY74	Cysteine synthase	Prevotella ruminicola	106.40	98.30	95.33	1.30	0.001	0.672	HIGH0
A0A1M6WB45	Starch phosphorylase	Prevotella ruminicola	97.47	102.03	100.47	0.42	0.001	0.003	LOW0
A0A1H9AWB6	TonB-linked outer membrane protein, SusC/RagA family	Prevotella bryantii	94.07	107.23	98.67	0.84	0.001	<0.0001	LOW0
A0A1H7IVN8	Phosphoenolpyruvate carboxykinase (ATP)	Pseudobutyrivibrio ruminis	106.70	97.57	95.73	1.59	0.002	0.395	HIGH0
C9RP04	Fructose-bisphosphate aldolase, class II	Fibrobacter succinogenes	102.90	98.70	98.43	0.67	0.002	0.193	HIGH0
W7UDV4	Pyruvate, phosphate dikinase	Ruminococcus flavefaciens	104.93	95.20	99.90	0.98	0.003	0.002	HIGH0
A0A1G5ID80	50S ribosomal protein L5	Ruminococcus bromii	96.63	99.30	104.07	1.08	0.004	0.140	LOW0
W7UL50	Nitrogen-fixing protein NifU	Ruminococcus flavefaciens	114.63	89.93	95.40	3.60	0.005	0.038	HIGH0
A0A1I0PQF4	50S ribosomal protein L31 type B	Prevotella aff. ruminicola	93.50	102.10	104.40	1.89	0.005	0.592	LOW0
W7UUA7	Cysteine synthase	Ruminococcus flavefaciens	109.07	95.70	95.23	2.49	0.005	0.230	HIGH0
H7ENF3	50S ribosomal protein L11	Treponema saccharophilum	94.50	101.23	104.23	1.68	0.006	0.911	LOW0
A0A1D9P2Q2	DNA-binding protein	Butyrivibrio hungatei	106.17	97.70	96.03	1.84	0.006	0.483	HIGH0
A0A1H7G1G5	GGGtGRT protein	Pseudobutyrivibrio ruminis	106.00	94.90	99.07	1.58	0.009	0.017	HIGH0
I5AT53	Elongation factor Tu	[Eubacterium] cellulosolvens	95.53	100.60	103.90	1.60	0.010	0.806	LOW0
I5ATH8	50S ribosomal protein L33	[Eubacterium] cellulosolvens	86.63	108.63	104.77	3.93	0.010	0.090	LOW0
A0A1T4L946	GGGtGRT protein	Eubacterium ruminantium	104.27	97.60	98.13	1.33	0.011	0.172	HIGH0
I5AUJ3	Phosphomannomutase	[Eubacterium] cellulosolvens	104.60	98.10	97.30	1.53	0.011	0.424	HIGH0
A0A1H3ZTM3	DUF4301 domain-containing protein	Prevotella ruminicola	106.00	96.90	97.10	1.95	0.012	0.241	HIGH0
C9RPJ7	Phosphoenolpyruvate carboxykinase [GTP]	Fibrobacter succinogenes	102.93	99.47	97.63	1.08	0.013	0.962	HIGH0
A0A1H7IZW1	O-acetylhomoserine (Thiol)-lyase	Pseudobutyrivibrio ruminis	108.70	93.90	97.37	2.66	0.013	0.072	HIGH0
A0A1H4EPE5	Branched-chain amino acid aminotransferase	Prevotella ruminicola	104.43	97.97	97.63	1.57	0.016	0.362	HIGH0
H7ELT1	Cysteine synthase	Treponema saccharophilum	106.77	97.00	96.27	2.46	0.018	0.402	HIGH0
G0VRR0	Band_7_1 domain-containing protein	Megasphaera elsdenii	103.57	97.57	98.87	1.21	0.019	0.106	HIGH0
A0A011UEZ1	Pyruvate-flavodoxin oxidoreductase	Ruminococcus albus	102.40	100.10	97.50	1.08	0.020	0.500	HIGH0
A0A2N0UJ77	Elongation factor G	Ruminococcus bromii	96.43	99.77	103.80	1.64	0.021	0.469	LOW0
A0A1I0P7E5	50S ribosomal protein L3	Prevotella aff. ruminicola	93.23	104.20	102.53	2.41	0.022	0.164	LOW0
W7UV65	50S ribosomal protein L14	Ruminococcus flavefaciens	95.73	100.47	103.83	1.90	0.024	0.788	LOW0
A0A1I0P5X6	30S ribosomal protein S11	Prevotella aff. ruminicola	90.57	107.53	101.90	3.29	0.027	0.062	LOW0
A0A1M6U920	30S ribosomal protein S5	Prevotella ruminicola	93.17	103.60	103.20	2.68	0.027	0.305	LOW0
A0A1D9P3D5	30S ribosomal protein S5	Butyrivibrio hungatei	91.67	100.60	107.73	3.90	0.027	0.727	LOW0
A0A1H3YTN0	Phosphate acetyltransferase	Prevotella ruminicola	103.47	98.00	98.53	1.35	0.028	0.242	HIGH0
A0A1T4JZ88	Acyl-CoA dehydrogenase	Eubacterium ruminantium	104.67	98.50	96.87	2.00	0.028	0.712	HIGH0
A0A1I0P683	30S ribosomal protein S5	Prevotella aff. ruminicola	95.83	103.87	100.27	1.39	0.029	0.027	LOW0
A0A1D9P124	Glycogen synthase	Butyrivibrio hungatei	104.80	97.03	98.20	1.88	0.031	0.200	HIGH0
A0A1H5TKQ7	TonB-linked outer membrane protein, SusC/RagA family	Prevotella ruminicola	97.30	102.37	100.37	0.96	0.032	0.045	LOW0
A0A0E3SKP3	60 kDa chaperonin	Methanosarcina barkeri	102.87	98.53	98.60	1.19	0.032	0.354	HIGH0
A0A1H6LAW0	Carboxypeptidase regulatory-like domain-containing protein	Prevotella ruminicola	95.70	103.03	101.27	1.65	0.033	0.129	LOW0
A0A011V0N9	Triosephosphate isomerase	Ruminococcus albus SY3	108.47	91.30	100.20	2.87	0.036	0.017	HIGH0
A0A1H5WW23	30S ribosomal protein S7	Prevotella ruminicola	96.07	105.00	98.93	1.17	0.036	0.003	LOW0
A0A011V181	50S ribosomal protein L6	Ruminococcus albus	91.87	102.53	105.63	3.81	0.037	0.765	LOW0
A0A1I0P6K5	50S ribosomal protein L14	Prevotella aff. ruminicola	94.23	103.83	101.97	2.35	0.038	0.181	LOW0
A0A1H5VAJ1	Fumarate hydratase class I	Prevotella ruminicola	104.63	98.57	96.80	2.18	0.038	0.767	HIGH0
A0A1T4MD15	Glyceraldehyde-3-phosphate dehydrogenase	Eubacterium ruminantium	103.57	98.23	98.20	1.57	0.039	0.404	HIGH0
I5AVX2	30S ribosomal protein S11	[Eubacterium] cellulosolvens	97.80	101.33	100.90	0.95	0.041	0.260	LOW0
A0A1H5SU14	Phosphoenolpyruvate carboxykinase (ATP)	Prevotella ruminicola	101.97	99.70	98.33	1.00	0.041	0.905	HIGH0
A0A1H7LBE7	Benzoyl-CoA reductase/2-hydroxyglutaryl-CoA dehydratase subunit, BcrC/BadD/HgdB	Pseudobutyrivibrio ruminis	102.83	100.03	97.13	1.53	0.042	0.619	HIGH0
A0A1D9P3R0	3-hydroxyacyl-CoA dehydrogenase	Butyrivibrio hungatei	108.47	95.03	96.50	3.67	0.042	0.278	HIGH0
D5EZ18	Uncharacterized protein	Prevotella ruminicola	96.00	101.27	102.77	1.94	0.042	0.765	LOW0
A0A1H7FPT3	50S ribosomal protein L21	Pseudobutyrivibrio ruminis	93.77	104.53	101.63	2.54	0.042	0.131	LOW0
A0A1H7FUZ2	Elongation factor G	Pseudobutyrivibrio ruminis	97.23	101.17	101.63	1.30	0.043	0.560	LOW0
A0A011V4S8	Glyceraldehyde-3-phosphate dehydrogenase	Ruminococcus albus SY3	105.93	95.33	98.77	2.38	0.044	0.098	HIGH0
A0A1G5IE48	50S ribosomal protein L4	Ruminococcus bromii	95.43	100.93	103.63	2.32	0.044	0.991	LOW0
A0A1H7KRN7	50S ribosomal protein L22	Pseudobutyrivibrio ruminis	90.73	106.67	102.60	3.89	0.046	0.149	LOW0
W7UV77	Twitching motility protein pilT	Ruminococcus flavefaciens	108.67	89.77	101.57	3.17	0.061	0.012	HIGH0
A0A1H4EAK7	50S ribosomal protein L1	Prevotella ruminicola	94.87	105.67	99.47	2.34	0.103	0.038	LOW0
D5EX68	Acyl carrier protein	Prevotella ruminicola	111.80	86.23	101.93	5.29	0.106	0.028	HIGH0
Q7MSE9	Pilin biogenesis	Wolinella succinogenes	109.00	89.73	101.30	4.29	0.122	0.038	HIGH0
A0A1I0P6T5	50S ribosomal protein L16	Prevotella aff. ruminicola	96.30	104.33	99.37	1.95	0.160	0.049	LOW0
A0A1H4CD71	Alpha-amylase	Prevotella ruminicola	98.70	102.57	98.77	0.70	0.398	0.005	LOW0
D5EUS6	50S ribosomal protein L16	Prevotella ruminicola	97.73	110.00	92.30	4.06	0.731	0.020	LOW0
G0VPW5	60 kDa chaperonin	Megasphaera elsdenii	101.73	95.27	103.00	1.91	0.913	0.022	HIGH0

## Discussion

Understanding microbial metabolism is a crucial step in the development of strategies to support and sustain maximal nutrient use efficiency in the rumen. Inclusion of proteomic techniques to articulate the underlying shifts in the rumen metaproteome is in its infancy, but gel-based protein fractionation from samples collected in static points of time highlight the breadth of protein identifications that can be achieved^15,27^. Using a combination of rumen-specific fractionation protocols with isobaric labeling techniques, the research reported herein is the first to outline the potential dynamic range of the rumen microbial metaproteome relative to first morning feeding. For this research, 47 composite database searches were completed, encompassing 19 microbial species and numerous strains. While this represents only a small fraction of the rumen microbiota, and further work must be done to ensure inclusive and accurate proportional representation of the rumen microbiota, this research encompasses the largest rumen metaproteomic search to date. Snelling and Wallace^27^ were unable to characterize the metaproteome of grazing dairy cattle due to impeding compounds on gels, however, they were able to distinguish 50 unique proteins derived from other ruminants such as lambs and beef cattle. Hart *et al*.^15^ highlighted the 25 most common protein families found within each individual cow and discussed phyla dominance but did not delineate proteomes of specific microbes.

In the present study, there were 43 variants of ribosomal proteins characterized within this research (16 30 s and 27 50 s) across 16 microbial species and is the most represented protein in this study. While some of these identified proteins may be redundant due to overlapping peptide sequences, approximately 20% of bacterial dry weight is made up of ribosomal proteins so it is not unexpected to see that these proteins are the majority of identified proteins^28^. These ribosomal proteins are subunits within a larger bacterial ribosome that serves in mRNA translation to protein, and each subunit serves a specific function. The smaller 30 s subunit decodes the mRNA strand while the larger 50 s subunit assists in peptide bonding of the specified amino acids. Another protein involved heavily in the central dogma, elongation factor Tu, was also a widely represented protein being characterized within 13 microbial species. This protein contributes to the continuous process of sequentially adding amino acids to a peptide chain. Overall, nearly all LOW0 proteins are translation-related. Ribosomal proteins (50 s and 30 s) and their associated variants were the most commonly affected proteins across sampling time with 53.5% of identified ribosomal proteins being affected by time of sampling, and 95.7% of the affected ribosomal proteins being represented in LOW0. Elongation factors (G, *R. flavefaciens*; Tu, *P. ruminis* and *R. bromii*) showed an increase in abundance relative to 0 h which also placed them in the LOW0 group.

To interpret the results, we broadly partitioned the rumen metaproteome into two conceptual categories, the first category being the above discussed general pathways of transcription and translation. The second category includes proteins with more specific roles in metabolic pathways. While it can be difficult to draw conclusions regarding the substrate or environmental drivers causing an increase in proteins related to translational processing beyond simply surmising that protein synthesis is likely increasing, there were many proteins identified in the current study that are key players in many specific metabolic pathways, highlighting possible shifts in more specific rumen functionality. In contrast to proteins grouped in LOW0, in HIGH0 there was largely a lack of representation of 30 s/50 s ribosomal proteins, elongation factors, and other translation-related proteins. Instead, there were shifts in proteins with more targeted functions, such as fumarate hydrolase, phosphoenol pyruvate carboxykinase, cysteine synthase, nitrogen-fixing protein NifU, GGGtGRT protein, pyruvate, phosphate dikinase, and glyceraldehyde-3-phosphate dehydrogenase (GAPDH). These results highlight the concept that rumen microbiota are independently reactive to their environment but yet are in synchrony with each other. Investigation of only the substrates or products within the rumen would not have likely yielded a comprehensive understanding of the individual microbial activities including the extent of synchronicity or similarity in protein profile across the different species within the rumen.

A consideration from the results is that the identified protein shifts illustrate that feeding protocol- and bunk management- induced fasting periods may quickly limit potential productivity of microbes. It is important to note that only 10.3% of analyzed proteins were affected by time of sampling, and could be due to the fact that cows were not fasted or subject to a more significant dietary perturbance or limitation beyond typical daily feeding schedule. Regardless, these results provide insight into the pathways and microbes more readily impacted by substrate-mediated suppression of microbial protein synthesis during non-eating periods^29^, and the impact that it may have on total microbial biomass production in the rumen and the consequent intestinal supply of microbial protein^30^.

Another aspect of rumen function highlighted from the current results is that nutrient deprivation may expose the fundamental metabolic pathways of specific microbes. Identifying the proteins in HIGH0 may give insight into pathways that specific microbes deem vital. As highlighted by^31^, identifying and exploiting the roles of independent microbes or groups of microbes is a current challenge. The inclusion of proteomics in study methodology can more broadly highlight pathways affected by a treatment or physiologic state rather than focusing on a small set of parameters to directly or indirectly assess ruminal changes and can identify metabolic shifts that do not result in a change in microbial diversity. The use of dietary models in combination with these proteomic techniques is also proposed to be a feasible method to better identify the basic roles of the rumen microbes, and what protein-mediated pathways the different microbes divert to in different nutrient scenarios or feed management protocols.

This research is the first published work to report the rumen metaproteome beyond static points in time and demonstrates how proteomic technology can provide a meaningful contribution to the characterization of microbial activity and protein-mediated pathway dynamics. The trial reported herein demonstrates that the rumen protein profile is dynamic and appears to be sensitive to lower nutrient availability. Furthermore, this research supports the hypothesis that inclusion of proteomic technology to characterize the rumen metaproteome and the impact of diet, health, and environment on rumen functionality can provide a useful contribution and further advance our research to maximize production efficiency.

## Methods

### Animals and maintenance

Samples were collected from three lactating Holstein dairy cows (207 ± 53.5 days in milk) housed at the Paul R. Miller Research Complex (The University of Vermont, Burlington, VT, USA). Cows were fed a nutritionally balanced dietary ration (diet chemical composition listed in Supplementary Table S3) *ad libitum* and were offered a total mixed ration twice daily (0630 h and 1430 h) and a diet supplement (high grain pellet) four times daily (0645 h, 1045 h, 1730 h, and 2300 h). All feed refusals were discarded prior to morning (0630 h) feeding daily. Cows had *ad libitum* access to water. Animal use and samplings methods performed in this trial were reviewed and approved by the Institutional Animal Care and Use Committee of the University of Vermont (Protocol #16-029) in accordance with the requirements of the Office of Laboratory Animal Welfare.

### Rumen sampling

As per sampling protocols previously described^32–35^, rumen fluid (RF) samples were collected from cows at 0630 h (0 h), 1030 h (4 h), and 1230 h (6 h) on day 1, 3, and 5 of a 5-day protocol. The 0 h samples were collected after morning refusals were collected but immediately prior to initial TMR offerings.

As per methods outlined by Steele *et al*.^35^, digesta grab samples were collected at each timepoint via a rumen cannula from beneath the fiber mat within the ventral sac of the rumen. A minimum of three digesta grab samples were collected per cow at each sampling point for a single representative sample per timepoint. Samples were immediately snap frozen in a dry-ice ethanol bath, transported on dry ice to the laboratory, and stored at −80 °C until processing.

### Rumen sample processing

For processing, RF samples were thawed overnight at 4 °C. Once thawed, samples were filtered through 4 layers of cheesecloth (Lion Services Inc., Charlotte, NC, USA), and filtered samples were composited within cow within timepoint across day for a representative sample of 0 h, 4 h, and 6 h for each cow prior to freezing at −80 °C. For centrifugation, the composited filtered samples were thawed on ice and centrifuged at 16,000 × *g* for 20 minutes at 4 °C. The resulting supernatant was discarded, and the remaining pellets were retained.

The collected pellets were lysed using both chemical and mechanical lysis methods based on both Snelling and Wallace^27^ and Yu and Morrison^36^ with modifications. Briefly, 1.5 mL of RIPA lysis buffer containing protease inhibitor (Pierce^TM^ Protease Inhibitor Mini Tablets, Thermo Scientific, Rockford, IL, USA) and a 5 mm stainless steel bead (Qiagen, Hilden, Germany) was added to each pelleted sample and samples were homogenized (TissueLyser II, Qiagen, Hilden, Germany) through six repetitions of lysis at 30 Hz for 30 seconds with a 3-min incubation on ice between each repetition, similar to the BeadBeater protocol reported by Luccitt *et al*.^37^. A subsample of each sample homogenate was pipetted into a clean tube and precipitated overnight at 4 °C in a lysis solution (6 M TCA, 80 mM DTT) (3:1 protein extract to TCA/DTT) similar to the protocol outlined by Snelling & Wallace^27^.

Following the overnight incubation, the samples were vortexed and centrifuged at 16,000 × *g* for 20 minutes at 4 °C and supernatants were discarded. The retained pellets were then washed four times as per methods of Snelling and Wallace^27^ with modifications by Song *et al*.^38^, where the retained pellets were washed in ice-cold 20% DMSO in acetone, incubated for 1 hour at −20 °C, and centrifuged at 10,000 × *g* for 5 minutes at 4 °C. The supernatants were then discarded, and the pellets were again washed using the same protocol. This wash protocol was then repeated twice more using 100% ice-cold acetone. After the final wash, the collected pellets were air dried and resuspended in phosphate buffered saline. A new 5 mm stainless steel ball was added to each sample before samples were homogenized in the TissueLyser for 30 seconds at 30 Hz. A universal control (UC) sample was generated by combining equal volumes from each of the 9 samples. Samples were stored at −80 °C until protein quantification of samples was performed using the bicinchonic acid assay (BCA) kit (Pierce, Rockford, IL, USA).

### TMT isobaric labeling, high pH reversed-phase peptide fractionation and liquid chromatography-tandem mass spectrometry (LC-MS/MS)

Quantified samples (85 μg) were then labeled using TMT Isobaric Tags as per manufacturer instructions (Thermo Scientific, Rockford, IL, USA). Labeling efficiency of each samples was verified to be more than 96% through preliminary MS analysis of individual samples. Equal volumes (75 μL) of each TMT-labeled sample was combined into a new tube and a 100 μL aliquot was vacuum dried to remove the triethyl ammonium bicarbonate (TEAB). The peptides were then fractionated using the high pH reversed-phase peptide fractionation kit (Thermo Scientific, Rockford, IL, USA) as per kit instructions resulting in 8 fractions for LC-MS/MS per original sample. One-tenth of each of the fractionated samples was dried down and resuspended in 2.5% formic acid (FA) in water and 2.5% acetonitrile (CH_3_CN). The LC-MS/MS analysis was carried out on the Q-Exactive Plus mass spectrometer coupled to an EASY-nLC 1200 (Thermo Scientific, Waltham, MA, USA) performed by the VGN Proteomics Facility (Burlington, VT, USA). Peptides were separated using a gradient of 2.5–35% CH_3_CN/0.1% FA over 60 min, 35–100% CH_3_CN/0.1% FA in 1 min and then 100% CH_3_CN/0.1% FA for 4 min, followed by an immediate return to 2.5% CH_3_CN/0.1% FA and a hold at 2.5% CH_3_CN/0.1% FA. The nanospray and data acquisition methods were completed per Scuderi *et al*.^39^. Briefly, samples were loaded onto a 100 μm × 500 mm capillary column packed with Halo C18 (2.7 μm particle size, 90 nm pore size, Michrom Bioresources, CA, USA) at a flow rate of 300 nL min^−1^. The column end was laser pulled to a ~3 μm orifice and packed with minimal amounts of 5um Magic C18AQ before packing with the 3-μm particle size chromatographic materials. Peptides were introduced into the mass spectrometer via a nanospray ionization source with a spray voltage of 2.0 kV. Mass spectrometry data was acquired in a data-dependent “Top 10” acquisition mode with lock mass function activated (*m/z* 371.1012; use lock masses: best; lock mass injection: full MS), in which a survey scan from *m/z* 350–1600 at 70, 000 resolution (AGC target 1e^6^; max IT 100 ms; profile mode) was followed by 10 higher-energy collisional dissociation (HCD) tandem mass spectrometry (MS/MS) scans on the most abundant ions at 35,000 resolution (AGC target 1e^5^; max IT 100 ms; profile mode). MS/MS scans were acquired with an isolation width of 1.2 *m/z* and a normalized collisional energy of 35%. Dynamic exclusion was enabled (peptide match: preferred; exclude isotopes: on; underfill ratio: 1%).

### Data and statistical analysis

Product ion spectra were searched using SEQUEST and Mascot through Proteome Discoverer 2.2 (Thermo Scientific, Waltham, MA, USA) against 47 composite databases encompassing strains of 19 microbial species downloaded on Nov. 30, 2018, Jan. 29, 2019, and Nov. 11, 2019, including *E. ruminantium* (UP000189857), *L. ruminis* (UP000001279), *T. bryantii* (UP000182360), *T. saccharophilum* (UP000003571), *F. succinogenes* (UP000000517), *M. barkeri 3* (UP000033066), *M. elsdenii DSM20460* (UP000010111), *O. formigenes HOxBLS* (UP000003973), *R. albus SY3* (UP000021369), *R. flavefaciens 007c* (UP000019365), *W. succinogenes* (UP000000422), *B. hungatei* MB2003 (UP000179284), *B. hungatei* XBD2006 (UP000183047), *B. hungatei* DSM 14810 (UP000184097), *P. aff. ruminicola* Tc2-24 (UP000199373), *P. bryantii* FB3001 (UP000182952), *P. bryantii* KHPX14 (UP000183264), *P. bryantii* TC1-1 (UP000216189), *P. bryantii* B14 (UP000004524; UP000183837), *P. ruminicola* (Bacteroides ruminicola) AR32 (UP000236735), *P. ruminicola* (Bacteroides ruminicola) ATCC 19189 (UP000183727), *P. ruminicola* (Bacteroides ruminicola) BPI-162 (UP000182287), *P. ruminicola* (Bacteroides ruminicola) BPI-34 (UP000184280), *P. ruminicola* (Bacteroides ruminicola) D31d (UP000182257), *P. ruminicola* (Bacteroides ruminicola) KHT3 (UP000184130), *P. ruminicola* ATCC 19189/JCM 8958/23 and ATCC 19189/JCM 8958/23 (UP000000927), *P. cellulosolvens* ATCC 35603/DSM 2933 (UP000036923), *P. ruminis* ACV-9 (UP000182321), *P. ruminis* JK10 (UP000224317), *P. ruminis* JK626 (UP000225889), *P. ruminis* DSM 9787 (UP000219563), *R. bromii* strain 5AMG (UP000233562), *R. bromii* AF15-36 (UP000283859), *R. bromii* AF21-10LB (UP000283293), *R. bromii* AF25-7LB (UP000286041), *R. bromii* AM32-13AC (UP000284544), *R. bromii* AM46-2BH (UP000285083), *R. bromii* ATCC 27255 (UP000233425), *R. bromii* CF01-14 (UP000284438), *R. bromii* L2-36 (UP000233570), *R. bromii* TM09-18AC (UP000262420), *R. bromii* TM09-5AC (UP000264375), *R. bromii* TM10-21 (UP000263282), *R. bromii* YE282 (UP000198616), *R. bromii* L2-63 (UP000240927), and *E. cellulosolvens* (UP000005753). All 9 raw files were searched against the database as one contiguous input file. Search parameters were as follows: (1) full trypsin enzymatic activity; (2) mass tolerance at 10 ppm and 0.02 Da for precursor ions and fragment ions, respectively; (3) dynamic modifications: oxidation on methionine (+15.995 Da); (4) dynamic TMT6plex modification on N-termini and lysine (+229.163 Da); and (5) static carbamidomethylation modification on cysteines (+57.021 Da). Percolator node was included in the workflow to limit the false positive (FP) rates to less than 1% in the data set. The relative abundances of TMT labeled peptides were quantified with the Reporter Ions Quantifier node in the Consensus workflow and parameters were set as follows: (1) both unique and razor peptides were used for quantification; (2) Reject Quan Results with Missing Channels: False; (3) Apply Quan Value Corrections: False; (4) Co-Isolation Threshold: 50; (5) Average Reporter S/N Threshold = 10; (6) “Total Peptide Amount” was used for normalization and (7) Scaling Mode was set “on Control Average”, so that the peptide abundances in the UC labels were set as 100 and the abundances in other channels were scaled accordingly. Non-normalized data is listed in Supplementary Table S4. The normalized scaled abundance values were used for subsequent statistical analyses. For any proteins identified that remained “uncharacterized”, either PANTHER Classification System^40^ was used to identify to protein name using the accession number, or the FASTA sequence was retrieved from UnitProt^41^ and searched through BLAST^42^ and the top hit protein was selected as the protein ID if above 99.0% matched identity. PROC IML in SAS was used to determine appropriate weighting for orthogonal contrasts to determine linear and quadratic effects of time. The PROC MIXED of SAS was then utilized to determine the effect of time on the dependent variable of protein. Effect of time of sampling was deemed significant if P < 0.050.

## Supplementary information

Supplementary dataset 1-4.
